# The Effect of Metformin on the Myocardial Tolerance to Ischemia-Reperfusion Injury in the Rat Model of Diabetes Mellitus Type II

**DOI:** 10.1155/2011/907496

**Published:** 2011-06-22

**Authors:** Ekaterina Kravchuk, Elena Grineva, Alekber Bairamov, Michael Galagudza, Timur Vlasov

**Affiliations:** ^1^Institute of Endocrinology, Almazov Federal Heart, Blood and Endocrinology Centre, Akkuratova Street 2, St. Petersburg 197341, Russia; ^2^Institute of Experimental Medicine, Almazov Federal Heart, Blood and Endocrinology Centre, Akkuratova Street 2, St. Petersburg 197341, Russia; ^3^Department of Pathophysiology, St. Petersburg Pavlov State Medical University, Lev Tolstoy Street 6/8, St. Petersburg 197022, Russia

## Abstract

In recent years, evidence has been accumulated that metformin, an antidiabetic drug in the biguanide class, in addition to its well-recognized glucose-lowering effect, can also reduce cardiovascular mortality in the patients with type 2 diabetes mellitus (T2DM). Besides, there are a few experimental studies on the possibility of the direct anti-ischemic effect of the drug in both type 1 diabetes mellitus and T2DM. In our study, myocardial tolerance to ischemia in rats with neonatal streptozotocin T2DM was investigated using the model of global ischemia-reperfusion of the isolated perfused heart. Metformin was administered i.p. at a dose of 200 mg/kg/day for 3 days prior to isolated heart perfusion. The results showed that both the infarct size and postischemic recovery of left ventricular function were not different between controls and metformin-treated animals. At the same time, the infarct size in the T2DM animals was significantly lower than that in the controls (24.4 ± 7.6% versus 45.0 ± 10.4%, resp., *P* < .01), indicative of the metabolic preconditioning in T2DM. It follows that the protocol of metformin administration used in this study had not elicited cardioprotective effect in animals with T2DM so that the different mechanism(s) may underlie the beneficial effect of metformin on cardiovascular complications in patients with T2DM which, however, would need further investigation.

## 1. Introduction

Ischemic heart disease (IHD) remains the leading cause of death in the patients with type 2 diabetes mellitus (T2DM) [1]. It seems apparent, therefore, that current pharmacotherapy of the patients with both IHD and T2DM should be aimed not only at glucose lowering but also at prevention of cardiovascular complications. In recent years, evidence has accumulated that metformin, the only antidiabetic drug available for clinical use in the biguanide class, in addition to its major glucose-lowering effect, can also exert several pleiotropic effects including beneficial changes in blood rheology, serum lipid profile, and putative anti-ischemic effects [2]. If this is the case, then metformin may have not a single but multiple mechanisms of action in cardiovascular disease, including cardio- and vasoprotection. This notion might be further supported by the results of prospective clinical trial UKPDS (United Kingdom Prospective Diabetes Study) [3]. In that trial, the mortality rates in patients with association of T2DM and excessive body weight were compared between the group receiving metformin and that receiving either sulfonylureas or insulin. The results showed that metformin reduced all-cause mortality by 36% while the mortalities associated with T2DM, myocardial infarction, and stroke were all decreased by 42, 39, and 41%, respectively. Furthermore, the precise mechanism (or mechanisms) of biguanide-induced reduction of cardiovascular risk is still poorly understood although some evidence is available for vasoprotective effect of metformin on the impaired vascular reactivity in the rats with T2DM owing probably to the increased activity of NO-synthase; antihypertensive effect of the drug was also demonstrated in spontaneously hypertensive rats [4, 5]. As far as the cardioprotective effect of metformin is concerned, it had been found that the addition of the drug to the perfusate resulted in the attenuation of left ventricular postischemic dysfunction (stunning) in the nondiabetic isolated rat heart model [6]. In the distinct study the infarct-limiting effect of metformin was demonstrated in mice with genetically determined T2DM after either single intraperitoneal injection or intraventricular administration during reperfusion [7]. Besides, regular oral administration of metformin increased myocardial tolerance to ischemia in animals with type I diabetes mellitus (T1DM) [8]. Molecular mechanisms of the above findings are currently under intense research. However, no information at all is available as yet on the possible cardioprotective effect of the repeated parenteral treatment with metformin in the animals with T2DM. 

The present study is aimed at the analysis of the protective effect of the repeated treatment with metformin on the global ischemia-reperfusion cardiac injury with use of the neonatal streptozotocin-induced T2DM rat model.

## 2. Materials and Methods

All experiments were performed in accordance with the “Guide for the Care and Use of Laboratory Animals” (publication no. [NIH] 85-23). 

### 2.1. Type 2 Diabetes Mellitus Model

The 3-4-day-old Wistar albino rats were used throughout the experiments. The model of streptozotocin-induced T2DM was used with i.p. injection of streptozotocin dissolved in the citrate buffer (pH = 5.5) at a dose of 65 mg/kg as described previously in [9]. Control animals received the same volume of the vehicle alone. The animals were grown on regular chow and water available *ad libitum*. 

### 2.2. Isolated Heart Perfusion

Detailed description of Langendorff isolated heart perfusion technique is provided elsewhere [10]. Briefly, the 10–12 week old animals were anesthetized with sodium pentobarbital (60 mg/kg), and the heart was excised via bilateral thoracotomy and perfused through the ascending aorta with Krebs-Henseleit buffer at the constant pressure of 85 mm Hg. The heart function was stabilized for 15 min followed by 30 min global ischemia finalized by 120 min reperfusion. During the experiments, left ventricular pressure was measured isovolumetrically with use of polyethylene balloon connected to the pressure transducer. The following parameters were monitored: left ventricular end-diastolic pressure (LVEDP), left ventricular developed pressure (LVDP), and heart rate (HR). Left ventricular pressure signal was processed with custom-made software. Besides, coronary flow rate (CFR) was measured by collection of perfusate outflow.

### 2.3. Experimental Protocol

The animals were allocated to four groups:

1. controls (*n* = 13): nondiabetic rats treated with intraperitoneal 0.9% sodium chloride solution injections for 3 days prior to the heart perfusion,
2. controls + metformin (CM; *n* = 12): nondiabetic rats treated with metformin (Nycomed, France) at a dose of 200 mg/kg i.p. for 3 days prior to the perfusion,
3. T2DM (*n* = 12): the animals with T2DM treated with intraperitoneal 0.9% sodium chloride solution injections for 3 days prior to the heart perfusion,
4. T2DM + metformin (T2DMM; *n* = 7): diabetic rats treated with metformin at a dose of 200 mg/kg i.p. for 3 days prior to the heart perfusion.

Hemodynamic parameters were registered at baseline, that is, 15 min before ischemia, immediately prior to ischemia, at 10, 20, and 30 min of ischemia, and at every 10 min of reperfusion. 

### 2.4. Infarct Size Determination

At the end of reperfusion, the hearts were rapidly cut into four equally spaced transverse slices. The slices were immersed in 1% solution of 2,3,5-triphenyltetrazolium chloride (TTC) for 15 min at 37°C. TTC-stained slices were photographed with digital camera Olimpus 2020 for further determination of TTC-negative (necrotic) area. After computer planimetry (Photoshop CS), the data were expressed as a ratio of necrotic area to the total slice one.

### 2.5. Statistical Analysis

Statistical differences in hemodynamic data, blood glucose concentration, and infarct size area were evaluated with STATISTICA software (ANOVA, Sheffe test). All data are presented as “mean ± standard deviation”. The differences were considered significant at *P* < .05. 

## 3. Results

### 3.1. Animal Body Weight and Blood Glucose Level

Regular weighing and blood glucose determination with use of Accu-Chek Performa glucometer (Roch, USA) were performed in all animals before heart perfusion. Just prior to the heart isolation, the control animals had an average weight of 187 ± 32 g compared to the significantly lower value (145 ± 13 g, *P* < .05) in the T2DM group. Blood glucose just prior to the heart isolation in control, CM, T2DM, and T2DMM groups was 6.4 ± 0.6, 5.8 ± 1.2, 9.5 ± 3.3 (*P* < .05 versus controls), and 8.2 ± 1.6 mmol/l (*P* < .05 versus controls), respectively. Metformin did not cause the significant decrease in blood glucose level in the T2DMM group, possibly because of relatively short duration of the treatment.

### 3.2. Cardiac Function

**Figure 1:**
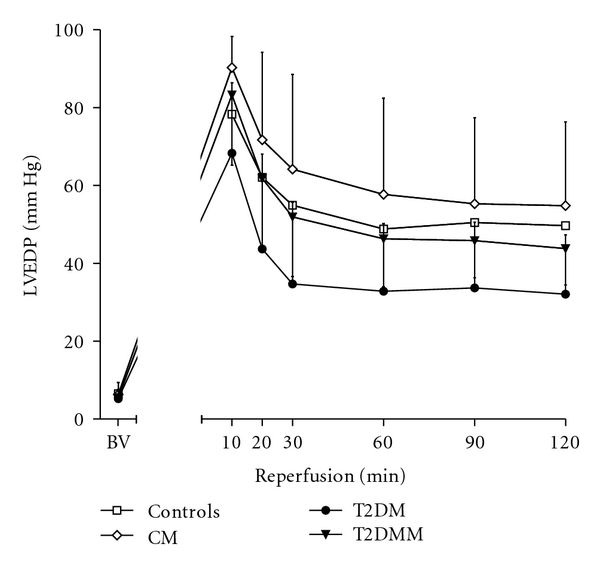
Left ventricular end-diastolic pressure (LVEDP) dynamics in the course of the experiments.

**Figure 2:**
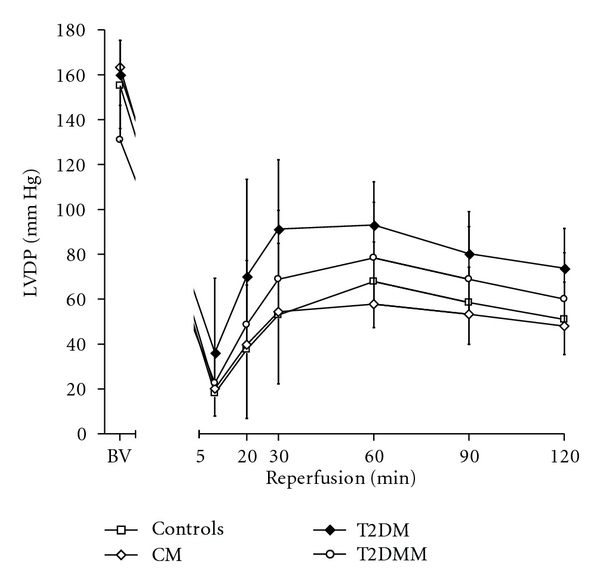
Left ventricular developed pressure (LVDP) dynamics in the course of the experiments.

**Figure 3:**
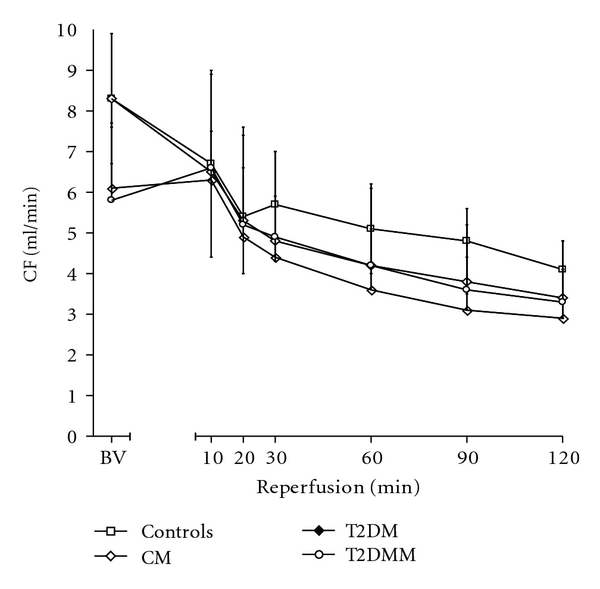
Coronary flow (CF) dynamics in the course of the experiments.

The baseline values of LVEDP, LVDP, CFR, and HR did not differ significantly among all groups (Figures 1, 2, and 3, Table 1). As to the dynamics, LVEDP increased to recover gradually with no difference between groups throughout reperfusion. However, there was a tendency towards lower values of LVEDP during reperfusion in the groups T2DM and T2DMM as compared to the controls. During reperfusion, LVDP and CF decreased in all groups of animals, with no significant difference between groups. The group values of HR during the experiments are shown in Table 1. Both preischemic and postischemic differences in HR within or between groups could be explained as due to chance. 

### 3.3. Myocardial Infarct Size

**Figure 4:**
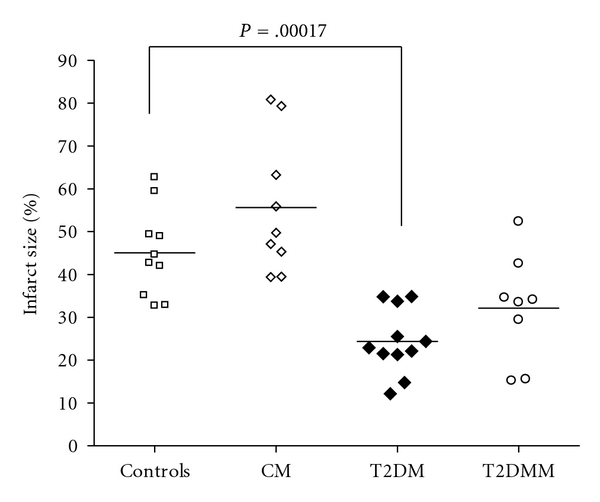
Infarct size data. Infarct size is significantly lower in the groups T2DM and T2DMM in comparison with the controls and CM.

Infarct size data are presented in Figure 4. In controls, infarct size expressed in percent (see above) averaged 45.0 ± 10.4%. Administration of metformin to healthy animals did not cause the significant change in infarct size (55.6 ± 15.8%). At the same time, infarct size was significantly lower in the animals with T2DM in comparison to the controls (24.4 ± 7.6%, *P* = .00017) while metformin treatment had no appreciable effect on this parameter in the animals with T2DM (37.7 ± 8.3%).

## 4. Discussion

The results of the present study demonstrate that metformin therapy at least with the dose used, route of administration, and the duration of treatment had no cardioprotective effect in both intact and T2DM diabetic animals. Furthermore, these data corroborate the concept of metabolic preconditioning based on the still paradoxical increase of myocardial tolerance to ischemia-reperfusion in the animals with both T1DM and T2DM [11].

The effect of metformin on the *in vivo* myocardial ischemia-reperfusion injury has been investigated by Calvert et al. in intact mice as well as in the transgenic T2DM animals [7]. In this study, only a single administration of metformin was used either 18 hours prior to ischemia or at the very beginning of reperfusion. The authors have concluded that metformin attenuated myocardial ischemic injury in either protocol of drug administration with no effect on blood glucose concentration. In the different study, the acute cardioprotective effect of a single metformin injection in the left ventricular cavity either prior or after 12 min global cardiac ischemia was examined in the intact isolated rat heart [6]. The authors demonstrated the significant improvement in coronary flow rate and also stroke volume during postischemic period in metformin-treated group as compared to the controls.

The beneficial cardiovascular effects of chronic metformin administration were shown in the rats with streptozotocin-induced T1DM; in particular, glucose-lowering effect and enhanced contractile response of the myocardium to increased preload were both observed after repeated oral treatment with metformin for 6 weeks [8]. In contrast, chronic oral administration of metformin for one month did not result in improved postischemic myocardial contractility in the isolated hearts from Zucker diabetic fatty rats [12] which is consistent with our findings. One possible explanation for the lack of cardioprotective effect of metformin given systemically prior to the isolated heart perfusion is drug washout which dramatically decreases tissue drug concentration at the time of ischemia and reperfusion. Suggestive of this, the more suitable models for investigation of metformin-mediated cardioprotection would be *in vivo* preparations or isolated heart experiments with addition of the drug into the perfusion fluid. Indeed, metformin pretreatment in rabbits subjected to two transient intravascular coronary occlusions separated by one week resulted in significant infarct size limitation after second period of ischemia [13].

Much less is known about the possible mechanisms of cardioprotective effect of the drug. Thus, the major therapeutic effects of metformin have been suggested to be because of its ability to activate adenosine monophosphate-activated protein kinase (AMPK) considered to be a common cellular “energy sensor” activating the energy-sparing metabolic processes resultant in the increased tolerance to hypoxia and ischemia [14, 15]. Noteworthy, metformin has been found to possess infarct-limiting effect when administered during reperfusion [7, 16]. This effect was associated with increased activation of AMPK, endothelial nitric oxide synthase, and phosphatidylinositol 3-kinase, the latter being functionally coupled to protein kinase B [16]. It seems that one of the most probable mechanisms underlying metformin-mediated myocardial protection against ischemia-reperfusion injury might include the prevention of mitochondrial permeability transition pore opening in the initial stage of reperfusion, which otherwise would lead to mitochondrial matrix swelling, energy wasting, and induction of apoptosis [16]. 

In conclusion, the data obtained show that, despite expectation, intraperitoneal treatment of diabetic rats with metformin for 3 days did not result in any cardioprotective effect. Most likely, the well-documented metformin-mediated reduction of cardiovascular mortality in the patients with T2DM is not directly related to the infarct size limitation but to a different mechanism, or mechanisms. Probably, such factors alone or in any combinations as metformin-induced reduction in the progression of atherosclerosis because of increased expression of antioxidant thioredoxin [17], attenuation of endothelial dysfunction [18], anti-inflammatory effect [19], inhibition of vascular smooth muscle cell proliferation [20], and decreased expression of adhesion molecules on the endothelial cells [21] may be involved. Further study with wide modifications of the experimental protocol, for example, the duration of ischemia, metformin dosage, schedule of metformin administration, or distinct approaches, may be needed to solve the issue.

## Figures and Tables

**Table 1:** Pre- and postischemic values of heart rate (HR, beats per minute) in different groups.

	Controls	Controls + metformin	T2DM	T2DM + metformin
Preischemic HR, beats per min	239 ± 48	254 ± 33	251 ± 32	249 ± 32
Postischemic HR:				
10 min	219 ± 39	235 ± 74	203 ± 37	278 ± 90
20 min	205 ± 34	252 ± 63	218 ± 22	286 ± 68
30 min	210 ± 45	262 ± 71	239 ± 51	268 ± 80
40 min	202 ± 59	268 ± 71	220 ± 34	270 ± 65
50 min	193 ± 45	233 ± 56	214 ± 36	266 ± 75
60 min	198 ± 50	249 ± 68	238 ± 39	269 ± 61
